# Supplementary material for: Birnessite: A New Oxidant for Green Rust Formation
Source: Materials (Basel). 2020 Aug 26;13(17):3777. doi: 10.3390/ma13173777 (PMC7504325; doi:10.3390/ma13173777)
Supplement: Supplementary file 1 [file materials-13-03777-s001.pdf]

materials

Supplementary Materials: Birnessite: A New Oxidant for Green Rust Formation

Amira Doggaz [1], Romain Coustel [1], Pierrick Durand [2], François Humbert [1] and Christian Ruby [1,*]

1 Université de Lorraine, CNRS, LCPME, 54600 Nancy, France ; amira.doggaz@univ-lorraine.fr (A.D.), romain.coustel@univ-lorraine.fr (R.C.), francois.humbert@univ-lorraine.fr (F.H.), christian.ruby@univ-lorraine.fr (C.R.)
2 Université de Lorraine, CNRS, CRM2, 54600 Nancy, France, pierrick.durand@univ-lorraine.fr
* Correspondence: christian.ruby@univ-lorraine.fr

Supplementary Information

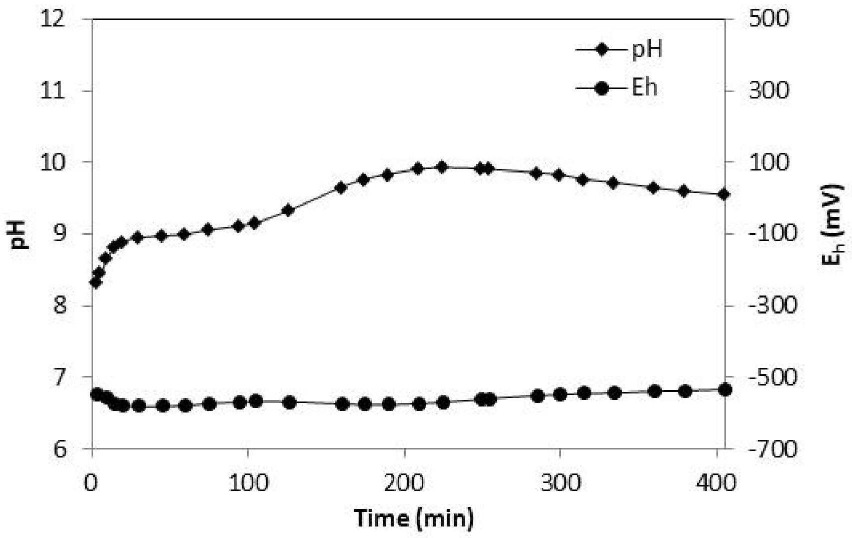

Figure 1. E_h and pH vs time for R = 0.6 and [Bir] = 5 g.L^{-1}, under N_2 blanketing. Birnessite added at t = 3 min.

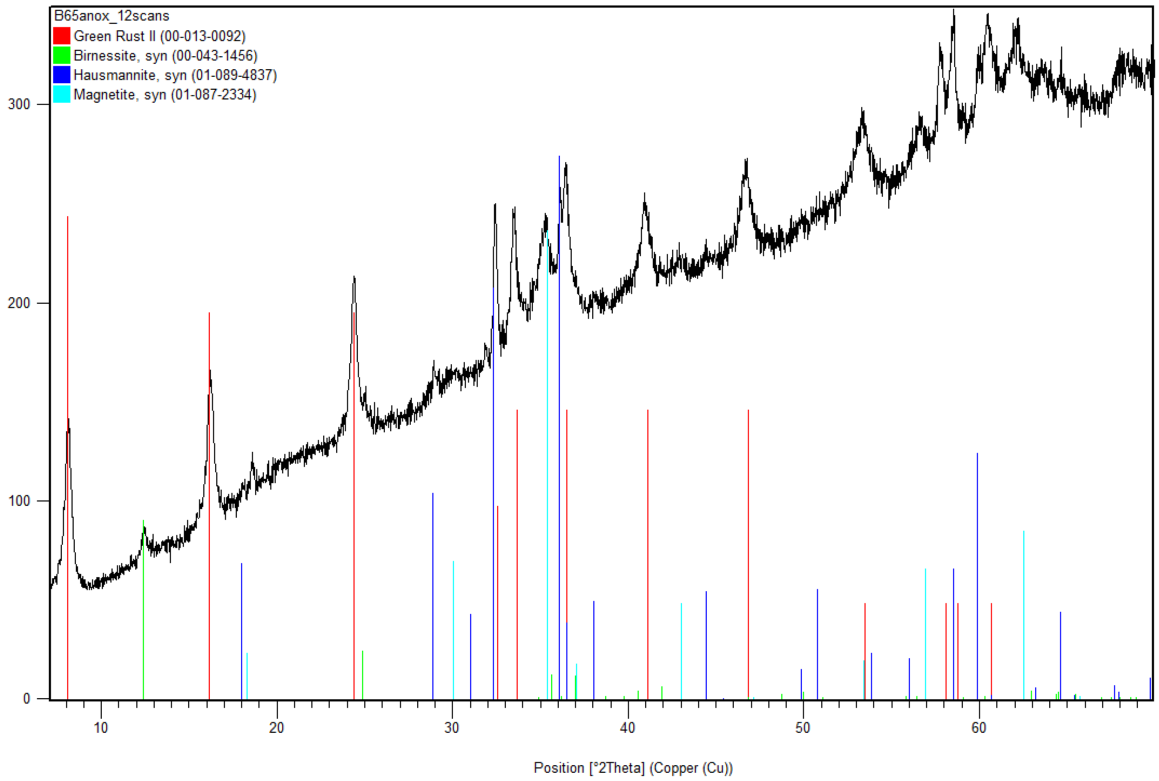

Figure 2. XRD patterns of R06B05IN2 (black). Tick marks of the patterns of birnessite (green, ICSD: 00-043-1456), hausmannite (blue, ICSD: 01-089-4837), green rust (red, ICSD: 00-013-0092) and magnetite (cyan, ICSD: 01-089-2355) are also shown as guidelines. The sample was prepared with R = 0.6, [Bir] = 5 g.L-1, under N2 blanketing. Solid product was withdrawn at t = 400 min (see Figure SI 1).

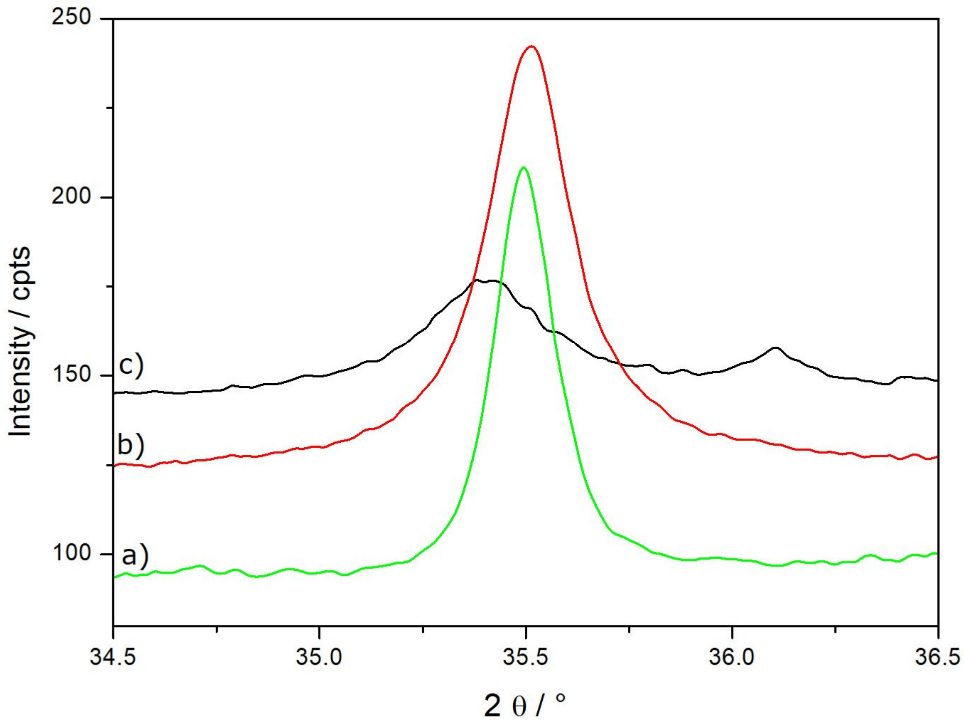

Figure 3. XRD patterns (**a**) R06F (green), (**b**) R06B02F (red), (**c**) R06B05F (black) samples. Zoom of Figure 2 in the region of (311) magnetite peak.

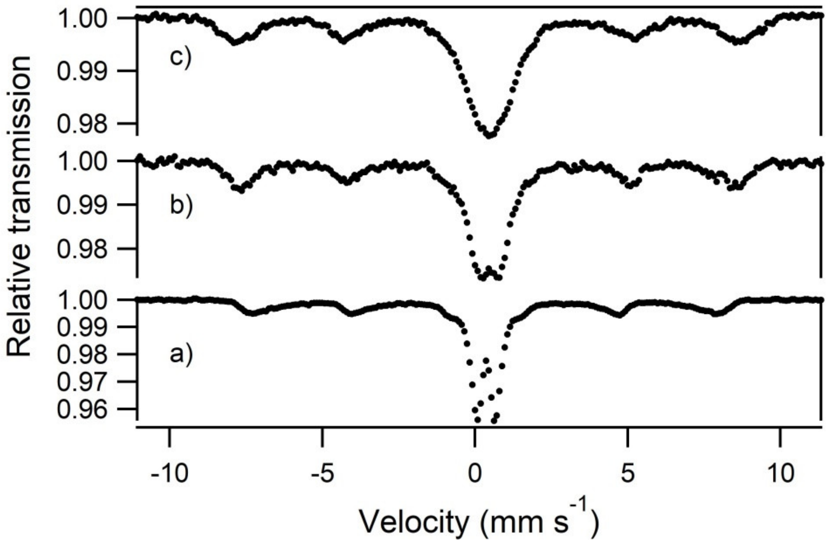

Figure 4. ^{57}Fe Mössbauer spectra of R06B05F recorded at (**a**) room temperature, (**b**) 140 K and (**c**) 77 K.

© 2020 by the authors. Licensee MDPI, Basel, Switzerland. This article is an open access article distributed under the terms and conditions of the Creative Commons Attribution (CC BY) license (http://creativecommons.org/licenses/by/4.0/).
